# Tetra­aqua­bis­(6-chloro­pyridine-3-carboxyl­ato-κ*O*)cobalt(II) tetra­hydrate

**DOI:** 10.1107/S160053681204319X

**Published:** 2012-10-20

**Authors:** Qiao-Hua Xia, Yue Zhang, Li Liu, Lin-Fang Shi, Bing Li

**Affiliations:** a College of Sciences, Zhejiang A&F University, Lin’an Hangzhou, Zhejiang 311300, People’s Republic of China; b College of Engineering, Zhejiang A&F University, Lin’an Hangzhou, Zhejiang 311300, People’s Republic of China

## Abstract

In the title compound, [Co(C_6_H_3_ClNO_2_)_2_(H_2_O)_4_]·4H_2_O, the Co^II^ cation is located on an inversion center and is coordinated by four water mol­ecules and two 6-chloro­pyridine-3-carboxyl­ate anions in a slightly distorted octa­hedral geometry. In the crystal, complex mol­ecules and lattice water mol­ecules are linked by O—H⋯O and O—H⋯N hydrogen bonds into a three-dimensional network.

## Related literature

For background and related structures, see: Long *et al.* (2007 ▶); Li *et al.* (2006 ▶).
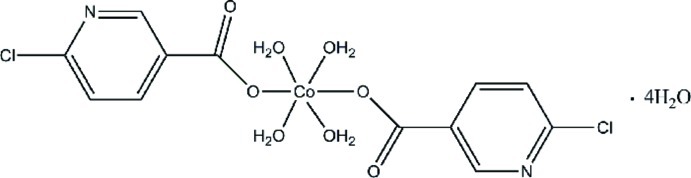


## Experimental

### 

#### Crystal data

[Co(C_6_H_3_ClNO_2_)_2_(H_2_O)_4_]·4H_2_O
*M* *_r_* = 516.15
Triclinic, 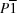

*a* = 7.0314 (14) Å
*b* = 7.3569 (15) Å
*c* = 11.564 (2) Å
α = 86.41 (3)°
β = 77.75 (3)°
γ = 64.80 (3)°
*V* = 528.7 (2) Å^3^
*Z* = 1
Mo *K*α radiation
μ = 1.13 mm^−1^
*T* = 293 K
0.42 × 0.37 × 0.18 mm

#### Data collection

Rigaku R-AXIS RAPID diffractometer
Absorption correction: multi-scan (*ABSCOR*; Higashi, 1995 ▶) *T*_min_ = 0.754, *T*_max_ = 0.862
5250 measured reflections
2394 independent reflections
2094 reflections with *I* > 2σ(*I*)
*R*_int_ = 0.065

#### Refinement

*R*[*F* ^2^ > 2σ(*F* ^2^)] = 0.029
*wR*(*F* ^2^) = 0.085
*S* = 1.16
2394 reflections
134 parameters
H-atom parameters constrained
Δρ_max_ = 0.45 e Å^−3^
Δρ_min_ = −0.48 e Å^−3^

### 

Data collection: *RAPID-AUTO* (Rigaku, 1998 ▶); cell refinement: *RAPID-AUTO*; data reduction: *CrystalStructure* (Rigaku/MSC, 2004 ▶); program(s) used to solve structure: *SHELXTL* (Sheldrick, 2008 ▶); program(s) used to refine structure: *SHELXTL*; molecular graphics: *SHELXTL*; software used to prepare material for publication: *SHELXTL*.

## Supplementary Material

Crystal structure: contains datablock(s) global, I. DOI: 10.1107/S160053681204319X/xu5631sup1.cif

Structure factors: contains datablock(s) I. DOI: 10.1107/S160053681204319X/xu5631Isup2.hkl

Additional supplementary materials:  crystallographic information; 3D view; checkCIF report

## Figures and Tables

**Table 1:** Hydrogen-bond geometry (Å, °)

*D*—H⋯*A*	*D*—H	H⋯*A*	*D*⋯*A*	*D*—H⋯*A*
O*W*1—H1*WA*⋯O1	0.86	1.81	2.644 (2)	162
O*W*1—H1*WB*⋯O*W*4	0.82	2.01	2.818 (2)	173
O*W*2—H2*WA*⋯O*W*4^i^	0.82	2.07	2.857 (2)	161
O*W*2—H2*WB*⋯O*W*3^i^	0.89	1.92	2.791 (2)	167
O*W*3—H3*WA*⋯N^ii^	0.85	2.00	2.842 (2)	172
O*W*3—H3*WB*⋯O1	0.81	1.95	2.763 (2)	176
O*W*4—H4*WA*⋯O*W*1^iii^	0.85	2.23	2.948 (2)	141
O*W*4—H4*WB*⋯O*W*3^i^	0.86	1.94	2.763 (2)	158

Symmetry codes: (i) 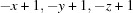; (ii) 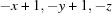; (iii) 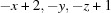.

**Fig. 1.**
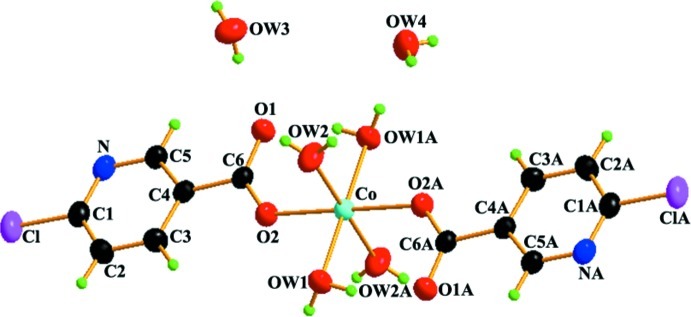
The molecular structure of the title complex, with 50% probability displacement ellipsoids for non-H atoms. [Symmetry codes: (A) 1 - x, -y, 1 - z.]

**Fig. 2.**
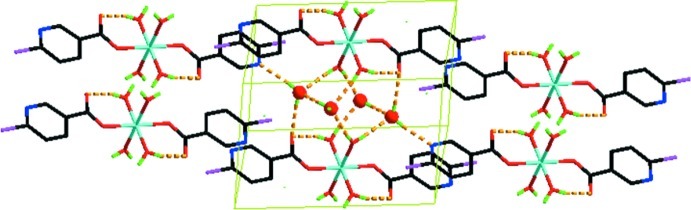
Crystal packing diagram for the title compound. All atoms are shown as isotropic spheres of arbitrary size. H atoms bonded to C atoms are omitted for clarity. The H-bonding interactions are shown as yellow dashed lines.

## supplementary crystallographic information

### Comment

In the crystal of the title compound, the Co(II) ion adopts a slightly distorted octahedral geometry and is located on a crystallographic inversion center. Four oxygen atoms from four coordination water molecules define the equatorial plane, while two oxygen atoms of two 6-chloro-3-carboxylate ligands occupy the axial sites (Figure 1). The Co—O bond lengths are in the range of 2.0723 (14)- 2.1162 (15) Å. The O—Co—O bond angles are 87.98 (6)–92.02 (6)° for the formally *cis* pairs of ligating atoms. The 6-chloropyridine-3-carboxylate carboxylate ligands are bound to the Co(II) ion in a monodentate mode through a carboxylate O atom. The three-dimensional supramolecular structure is formed by hydrogen bonds between six strong inter-molecular O—H···O and O—H···N hydrogen-bonding interactions and by additional intra-molecular O—H···O hydrogen bonds (Figure 2).

### Experimental

All commercially obtained reagent grade chemicals were used without further purication. A mixture of cobalt acetate tetrahydrate (0.4062 g) and 6-chloronicotinic acid (0.1310 g) were added into 20 ml water with 8 drops of 0.1 mol/*L* sodium hydroxide solution, and then stirred for 30 min. Finally, 5 ml 95% ethanol carefully layered above-mentioned solution in glass tube. After 1 day large pink platelet of the title compound suitable for X-ray diffraction were obtained.

### Refinement

H atoms bonded to C atoms were introduced in calculated positions and refined using a riding model [C—H = 0.93 Å and *U*_iso_(H) = 1.2U_eq_(C) for aromatic H atoms. H atoms belonging to water molecules were found in difference Fourier map and refined isotropically.

### Crystal data

**Table:**

[Co(C_6_H_3_ClNO_2_)_2_(H_2_O)_4_]·4H_2_O	*Z* = 1
*M**_r_* = 516.15	*F*(000) = 265
Triclinic, *P*1	*D*_x_ = 1.621 Mg m^−^^3^
Hall symbol: -P 1	Mo *K*α radiation, λ = 0.71073 Å
*a* = 7.0314 (14) Å	Cell parameters from 2394 reflections
*b* = 7.3569 (15) Å	θ = 3.1–27.5°
*c* = 11.564 (2) Å	µ = 1.13 mm^−^^1^
α = 86.41 (3)°	*T* = 293 K
β = 77.75 (3)°	Platelet, pink
γ = 64.80 (3)°	0.42 × 0.37 × 0.18 mm
*V* = 528.7 (2) Å^3^	

### Data collection

**Table:**

Rigaku R-AXIS RAPID diffractometer	2394 independent reflections
Radiation source: fine-focus sealed tube	2094 reflections with *I* > 2σ(*I*)
Graphite monochromator	*R*_int_ = 0.065
ω scans	θ_max_ = 27.5°, θ_min_ = 3.1°
Absorption correction: multi-scan (*ABSCOR*; Higashi, 1995)	*h* = −9→8
*T*_min_ = 0.754, *T*_max_ = 0.862	*k* = −9→9
5250 measured reflections	*l* = −15→15

### Refinement

**Table:**

Refinement on *F*^2^	Secondary atom site location: difference Fourier map
Least-squares matrix: full	Hydrogen site location: inferred from neighbouring sites
*R*[*F*^2^ > 2σ(*F*^2^)] = 0.029	H-atom parameters constrained
*wR*(*F*^2^) = 0.085	*w* = 1/[σ^2^(*F*_o_^2^) + (0.0248*P*)^2^ + 0.0893*P*] where *P* = (*F*_o_^2^ + 2*F*_c_^2^)/3
*S* = 1.16	(Δ/σ)_max_ = 0.001
2394 reflections	Δρ_max_ = 0.45 e Å^−^^3^
134 parameters	Δρ_min_ = −0.48 e Å^−^^3^
0 restraints	Extinction correction: *SHELXTL* (Sheldrick, 2008), Fc^*^=kFc[1+0.001xFc^2^λ^3^/sin(2θ)]^-1/4^
Primary atom site location: structure-invariant direct methods	Extinction coefficient: 0.356 (12)

### Special details

**Table:**

Geometry. All esds (except the esd in the dihedral angle between two l.s. planes) are estimated using the full covariance matrix. The cell esds are taken into account individually in the estimation of esds in distances, angles and torsion angles; correlations between esds in cell parameters are only used when they are defined by crystal symmetry. An approximate (isotropic) treatment of cell esds is used for estimating esds involving l.s. planes.
Refinement. Refinement of *F*^2^ against ALL reflections. The weighted *R*-factor *wR* and goodness of fit *S* are based on *F*^2^, conventional *R*-factors *R* are based on *F*, with *F* set to zero for negative *F*^2^. The threshold expression of *F*^2^ > σ(*F*^2^) is used only for calculating *R*-factors(gt) *etc*. and is not relevant to the choice of reflections for refinement. *R*-factors based on *F*^2^ are statistically about twice as large as those based on *F*, and *R*- factors based on ALL data will be even larger.

### Fractional atomic coordinates and isotropic or equivalent isotropic displacement parameters (Å^2^)

**Table:**

	*x*	*y*	*z*	*U*_iso_*/*U*_eq_	
Co	0.5000	0.0000	0.5000	0.02745 (15)	
Cl	0.00669 (8)	0.29228 (8)	−0.14887 (5)	0.04646 (18)	
O1	0.6093 (2)	0.1896 (2)	0.23682 (12)	0.0371 (3)	
O2	0.3932 (2)	0.0600 (2)	0.34210 (12)	0.0362 (3)	
OW1	0.77755 (19)	0.0456 (2)	0.42492 (13)	0.0368 (3)	
H1WA	0.7475	0.0959	0.3580	0.055*	
H1WB	0.8053	0.1204	0.4606	0.055*	
OW2	0.3261 (2)	0.3039 (2)	0.55709 (14)	0.0460 (4)	
H2WA	0.2602	0.4021	0.5201	0.069*	
H2WB	0.3531	0.3527	0.6166	0.069*	
OW3	0.5269 (2)	0.5885 (2)	0.26396 (14)	0.0476 (4)	
H3WA	0.5745	0.6384	0.2029	0.071*	
H3WB	0.5535	0.4718	0.2526	0.071*	
OW4	0.8332 (2)	0.3212 (2)	0.55760 (15)	0.0482 (4)	
H4WB	0.7442	0.3244	0.6224	0.072*	
H4WA	0.9634	0.2619	0.5653	0.072*	
N	0.3076 (2)	0.2781 (2)	−0.04625 (14)	0.0331 (4)	
C1	0.1321 (3)	0.2467 (3)	−0.02902 (17)	0.0320 (4)	
C2	0.0458 (3)	0.1820 (3)	0.07433 (18)	0.0361 (4)	
H2A	−0.0791	0.1638	0.0813	0.043*	
C3	0.1507 (3)	0.1451 (3)	0.16737 (18)	0.0347 (4)	
H3A	0.0989	0.0992	0.2384	0.042*	
C4	0.3362 (3)	0.1776 (2)	0.15307 (16)	0.0275 (4)	
C5	0.4056 (3)	0.2444 (3)	0.04594 (17)	0.0312 (4)	
H5A	0.5279	0.2678	0.0366	0.037*	
C6	0.4549 (3)	0.1400 (2)	0.25230 (16)	0.0277 (4)	

### Atomic displacement parameters (Å^2^)

**Table:**

	*U*^11^	*U*^22^	*U*^33^	*U*^12^	*U*^13^	*U*^23^
Co	0.0297 (2)	0.0310 (2)	0.0229 (2)	−0.01422 (15)	−0.00582 (13)	0.00395 (15)
Cl	0.0562 (3)	0.0444 (3)	0.0377 (3)	−0.0138 (2)	−0.0237 (2)	0.0020 (3)
O1	0.0387 (6)	0.0463 (8)	0.0343 (8)	−0.0251 (6)	−0.0107 (6)	0.0093 (7)
O2	0.0438 (7)	0.0453 (7)	0.0270 (7)	−0.0250 (6)	−0.0123 (5)	0.0102 (6)
OW1	0.0376 (6)	0.0474 (8)	0.0324 (7)	−0.0245 (6)	−0.0085 (5)	0.0051 (6)
OW2	0.0636 (9)	0.0306 (7)	0.0398 (9)	−0.0127 (7)	−0.0177 (7)	−0.0007 (7)
OW3	0.0655 (9)	0.0427 (8)	0.0366 (8)	−0.0288 (7)	−0.0013 (7)	−0.0007 (7)
OW4	0.0412 (7)	0.0517 (9)	0.0476 (9)	−0.0152 (7)	−0.0116 (7)	0.0065 (8)
N	0.0375 (8)	0.0326 (8)	0.0254 (8)	−0.0124 (7)	−0.0047 (6)	0.0039 (7)
C1	0.0360 (8)	0.0247 (8)	0.0290 (10)	−0.0051 (7)	−0.0091 (7)	−0.0034 (8)
C2	0.0313 (8)	0.0437 (10)	0.0355 (11)	−0.0173 (8)	−0.0081 (8)	0.0013 (9)
C3	0.0342 (8)	0.0430 (10)	0.0288 (10)	−0.0201 (8)	−0.0027 (7)	0.0028 (9)
C4	0.0300 (8)	0.0241 (8)	0.0246 (9)	−0.0087 (7)	−0.0034 (7)	−0.0003 (7)
C5	0.0311 (8)	0.0331 (9)	0.0284 (9)	−0.0137 (7)	−0.0046 (7)	0.0036 (8)
C6	0.0312 (8)	0.0237 (8)	0.0252 (9)	−0.0090 (7)	−0.0048 (7)	0.0002 (7)

### Geometric parameters (Å, º)

**Table:**

Co—O2^i^	2.0717 (14)	OW3—H3WB	0.8101
Co—O2	2.0717 (14)	OW4—H4WB	0.8629
Co—OW2	2.1078 (15)	OW4—H4WA	0.8520
Co—OW2^i^	2.1078 (15)	N—C1	1.322 (2)
Co—OW1	2.1157 (14)	N—C5	1.344 (2)
Co—OW1^i^	2.1157 (14)	C1—C2	1.375 (3)
Cl—C1	1.7379 (19)	C2—C3	1.380 (3)
O1—C6	1.261 (2)	C2—H2A	0.9300
O2—C6	1.250 (2)	C3—C4	1.398 (3)
OW1—H1WA	0.8642	C3—H3A	0.9300
OW1—H1WB	0.8153	C4—C5	1.373 (3)
OW2—H2WA	0.8246	C4—C6	1.504 (2)
OW2—H2WB	0.8853	C5—H5A	0.9300
OW3—H3WA	0.8466		
			
O2^i^—Co—O2	180.0	H3WA—OW3—H3WB	111.9
O2^i^—Co—OW2	88.51 (6)	H4WB—OW4—H4WA	112.2
O2—Co—OW2	91.49 (6)	C1—N—C5	116.27 (18)
O2^i^—Co—OW2^i^	91.49 (6)	N—C1—C2	125.08 (17)
O2—Co—OW2^i^	88.51 (6)	N—C1—Cl	115.68 (16)
OW2—Co—OW2^i^	180.0	C2—C1—Cl	119.24 (14)
O2^i^—Co—OW1	88.00 (6)	C1—C2—C3	117.79 (17)
O2—Co—OW1	92.00 (6)	C1—C2—H2A	121.1
OW2—Co—OW1	91.76 (7)	C3—C2—H2A	121.1
OW2^i^—Co—OW1	88.24 (7)	C2—C3—C4	118.94 (19)
O2^i^—Co—OW1^i^	92.00 (6)	C2—C3—H3A	120.5
O2—Co—OW1^i^	88.00 (6)	C4—C3—H3A	120.5
OW2—Co—OW1^i^	88.24 (7)	C5—C4—C3	117.88 (17)
OW2^i^—Co—OW1^i^	91.76 (7)	C5—C4—C6	121.45 (15)
OW1—Co—OW1^i^	180.00 (3)	C3—C4—C6	120.67 (18)
C6—O2—Co	128.85 (12)	N—C5—C4	124.03 (16)
Co—OW1—H1WA	100.8	N—C5—H5A	118.0
Co—OW1—H1WB	117.8	C4—C5—H5A	118.0
H1WA—OW1—H1WB	110.1	O2—C6—O1	125.95 (17)
Co—OW2—H2WA	129.2	O2—C6—C4	116.96 (15)
Co—OW2—H2WB	121.6	O1—C6—C4	117.09 (17)
H2WA—OW2—H2WB	105.8		

Symmetry code: (i) −*x*+1, −*y*, −*z*+1.

### Hydrogen-bond geometry (Å, º)

**Table:**

*D*—H···*A*	*D*—H	H···*A*	*D*···*A*	*D*—H···*A*
O*W*1—H1*WA*···O1	0.86	1.81	2.644 (2)	162
O*W*1—H1*WB*···O*W*4	0.82	2.01	2.818 (2)	173
O*W*2—H2*WA*···O*W*4^ii^	0.82	2.07	2.857 (2)	161
O*W*2—H2*WB*···O*W*3^ii^	0.89	1.92	2.791 (2)	167
O*W*3—H3*WA*···N^iii^	0.85	2.00	2.842 (2)	172
O*W*3—H3*WB*···O1	0.81	1.95	2.763 (2)	176
O*W*4—H4*WA*···O*W*1^iv^	0.85	2.23	2.948 (2)	141
O*W*4—H4*WB*···O*W*3^ii^	0.86	1.94	2.763 (2)	158

Symmetry codes: (ii) −*x*+1, −*y*+1, −*z*+1; (iii) −*x*+1, −*y*+1, −*z*; (iv) −*x*+2, −*y*, −*z*+1.
